# Academic excellence: A comprehensive investigation of medical students′ study habits, strategies, and sources in medical colleges of Peshawar, Pakistan

**DOI:** 10.12669/pjms.40.11.9502

**Published:** 2024-12

**Authors:** Muhammad Osama, Afaq Saeed, Muhammad Raza ul Haq, Mallahat Abdul Baseer

**Affiliations:** 1 Muhammad Osama Intern at the Department of Internal Medicine, Khyber Teaching Hospital, University Road, Peshawar, Pakistan; 2 Afaq Saeed House Officer Intern, Khyber Teaching Hospital, University Road, Peshawar, Pakistan; 3 Muhammad Raza Ul Haq House Officer, Intern, Khyber Teaching Hospital, University Road, Peshawar, Pakistan; 4 Mallahat Abdul Baseer, Final Year MBBS, Khyber Medical College, Peshawar, Pakistan

**Keywords:** Academic Excellence, Medical Students, Study Habits, Study Strategies

## Abstract

**Background & objective:**

It’s no secret that medical students have access to a plethora of medical literature, which can make it challenging to determine the right sources and how to cover them effectively using various study habits and strategies. Our objective was to determine the association of study habits (time and location), study duration, study strategies, and study sources with academic performance.

**Methods:**

It was a cross-sectional study conducted in six Medical Colleges in Peshawar, Pakistan between 13^th^ April 2023 to 13^th^ Jun 2023. Total 138 students were selected for the study. A well-structured questionnaire was used for data collection. Participants were from both genders and in both public and private sector medical colleges. First-year students and students from medical colleges outside of Peshawar were excluded from the study. SPSS (Version 20.0) was used for data analysis.

**Results:**

The study revealed that daily study hours (during normal days), residence status, library study, morning study timings, active recall, MCQS solving, and watching online videos made a significant association with academic performance (P < 0.05). Pearson’s correlation showed a positive and statistically significant association between daily study hours (during normal days), study strategies, and academic performance (p < 0.05). The multiple regression model explained 30.5% of the variance in academic performance.

**Conclusion:**

This study established a noteworthy link between academic performance and various factors, including daily study hours, strategies, and specific study sources. Notably, morning study sessions, active recall, MCQs-based strategies, and online video resources showed significant associations with enhanced academic performance.

## INTRODUCTION

Worldwide, millions of students strive to get into medical schools, but only the best-performing students can do so. Medical schools present a challenging environment, as students face an enormous level of information and are inundated by the number of resources available for learning. Good study habits and proper resources are essential to achieving academic excellence.^1^ The clear link between different study habits, and resources with academic performance is much more complex and needs to be fully elucidated.

Many studies have identified different factors that can lead to higher or lower academic achievement. An Irish study found that prioritizing one’s work and studying in an organized manner are associated with higher academic outcomes. In contrast, a superficial approach to studying was associated with poor outcomes.^2^ A study by West and Sadoski found that two skills: time management and self-testing, bore better academic outcomes.^3^ Sayer M et al. report some unique findings in their study, stating that domestic, financial, and emotional problems primarily lead to poor academic outcomes.^4^ A Saudi Arabian study completely contradicts the findings of Al Shawwa et al., claiming that financial and domestic problems had no impact on academic achievement; they also found that the source of studying had no impact on academic achievement.^5^

We have conducted this study because of a lack of published data locally. Also, every individual study’s results are different from the other’s study results. Our target was to determine the association between the study habits, methods, and sources with the academic performance of the MBBS students. This study will be helpful to many students in modifying their methods, sources, and habits.

## METHODS

It was a cross-sectional study conducted between 13th April 2023 to 13th Jun 2023 at six public and private medical colleges in Peshawar, Pakistan. Informed consent was taken from all the participants before the study. **STROBE** guidelines were followed in the preparation of this manuscript.

### Ethical approval:

It was obtained from The Institutional Research and Ethical Review Board (IREB) of Khyber Medical College (KMC), Peshawar (Ref no. 221/DME/KMC. dated: April 13, 2023).

### Study participants and sampling technique:

A sample size of 138 was calculated utilizing the online sample size calculator Calculator.net (Available at: https://www.calculator.net/sample-size-calculator.html). The confidence interval (CI) was kept at 95%, the margin of error at 5% and a population proportion of 10% based on a previous study conducted in Kermanshah-Iran.^6^ Participants that were included in this research were studying in both public and private sector medical colleges, were from both genders, and were enrolled in 2^nd^ to 5^th^ year MBBS classes. Additionally, students enrolled in disciplines other than MBBS or from a medical college outside Peshawar were excluded. A convenience sampling technique was employed for the selection of participants.

### Questionnaire:

A well-structured questionnaire was developed through the online platform Google Forms. Participants were asked about their basic demographic characteristics, including their college name, age, gender, year of study, and residence. Performance in the last professional exam was considered as their academic performance. The questionnaire includes questions about daily study hours (during normal routine as well as exam routine), study location, study strategies, and study sources. Lynn criteria were utilized for face validation with a criterion threshold of 0.80 by six subject experts. After developing the questionnaire, a pilot study was conducted for content validity in which responses from 10 students were recorded. Necessary changes were made to the questionnaire after it. To minimize bias, these 10 students were later on excluded from data analysis.

### Data analysis:

The collected data were analyzed through SPSS software (IBM Corp. Released 2011. IBM SPSS Statistics for Windows, Version 20.0. Armonk, NY: IBM Corp.) All the qualitative variables were presented in the form of frequency and percentages while the quantitative variables were presented as mean ± SD. To draw a comparison between variables chi-square test, Pearsons’s correlation, and multiple regression analysis were used. *P* < 0.05 was considered significant.

### Questionnaire link:

https://docs.google.com/forms/d/e/1FAIpQLSfEMjK4Jtl2GV-7w5pKMOY9pjtEc51rEhxo9iXKv71IN5uiAw/viewform?vc=0&c=0&w=1&flr=0

## RESULTS

The demographic characteristics of the study participants are shown in Table-I. Out of 138, 84 students (60.8%) were from public sector medical colleges of Peshawar and 54 (39.13%) were from the private sector. In terms of academic performance, 22 (15.9%) had below-average scores i.e., ≤ 70% marks, 74 (53.6%) had average scores i.e., 70-80% marks, and 42 (18.17%) had above-average scores i.e., ≥ 80% marks.

**Table-I:** Demographic characteristics of students.

College Name	n	Age (M ± SD)	Gender		Year of Study				Residence	

			Male	Female	2nd	3rd	4th	5th	Day Scholar	Hostelite
KMC	55	23±2	34	21	12	6	16	21	30	25
KGMC	29	23±2	0	29	3	3	12	11	12	17
PMC	22	22±1	6	16	0	20	2	0	8	14
RMC	14	21±1	7	7	10	2	0	2	8	6
Kabir MC	13	23±2	12	1	3	1	0	9	8	5
NWSM	5	21±1	4	1	4	0	0	1	4	1
Total (N)	138	22.37±1.57	64	74	32	32	31	43	70	68
Percentages	100		46.37	53.62	23.18	23.18	22.46	31.15	50.72	49.27

***Notes:*** KMC = Khyber medical college, KGMC = Khyber girls’ medical college, PMC = Peshawar medical college, RMC = Rehman medical college, Kabir MC = Kabir medical college, NWSM =North west school of Medicine.

The majority of the students (72, 52.17%) had the habit of studying in the morning. Similarly, most of them (76, 55.07%) were studying at home. The most common way of studying was highlighting the text (89, 64.49%) while textbooks were found as the most common study source (134, 97.10%), (Table-II).

**Table-II:** Study habits association with academic performance using chi-square test.

Variables			^a^Percentage marks			Total		P- value

			60-70% (n)	70-80% (n)	80+% (n)	n	%	
Gender	Male		15	31	18	64	46.37	0.082
	Female		7	43	24	74	53.62
Residence	Day Scholar		7	34	29	70	50.72	0.009
	Hostelite		15	40	13	68	49.27
Daily study hours (Normal days)	1-3hrs		19	61	7	87	63.04	0.000^b^
	4-5hrs		3	11	29	43	31.15	
	6-7hrs		0	1	3	4	2.8	
	8-10hrs		0	1	3	4	2.8	
Daily study hours (Exam days)	1-5hrs		3	4	2	9	6.52	0.171^b^
	6-10hrs		7	34	25	66	47.82	
	11-15hrs		8	28	14	50	36.23	
	16-20hrs		4	8	1	13	9.42	
Study location	Home	Yes	11	44	21	76	55.07	0.538
		No	11	30	21	62	44.92
	Library	Yes	4	30	6	40	28.98	0.005
		No	18	44	36	98	71.01
	Hostel	Yes	11	30	21	62	44.92	0.538
		No	11	44	21	76	55.07
Study Timings	Morning	Yes	9	33	30	72	52.17	0.011
		No	13	41	12	66	47.82
	Afternoon	Yes	9	32	16	57	41.30	0.863
		No	13	42	26	81	58.69
	Evening	Yes	6	21	13	40	28.98	0.94
		No	16	53	29	98	71.01
	Night	Yes	4	21	7	32	23.18	0.296
		No	18	53	35	106	76.81
Study Strategies	Active recall	Yes	3	17	24	44	31.88	0.0001
		No	19	57	18	94	68.11
	Repeated text reading	Yes	12	49	23	84	60.86	0.384
		No	10	25	19	54	39.13
	Highlighting	Yes	16	51	22	89	64.49	0.137
		No	6	23	20	49	35.50
	MCQS solving	Yes	3	22	21	46	33.33	0.009
		No	19	52	21	92	66.66
	Spaced repetition	Yes	8	22	19	49	35.50	0.244
		No	14	52	23	89	64.49
Study Sources	Standard textbooks	Yes	21	71	42	134	97.10	0.227^b^
		No	1	3	0	4	2.89
	MCQS books	Yes	8	37	21	66	47.82	0.502
		No	14	37	21	72	52.17
	Online videos	Yes	12	50	36	98	71.01	0.021
		No	10	24	6	40	28.98
	Teachers’ slides	Yes	9	26	13	48	34.78	0.726
		No	13	48	29	90	65.21
	Journals’ articles	Yes	1	0	1	2	1.4	0.190^b^
		No	21	74	41	134	97.10

***Notes:***

a = During the last professional exam,

b = Likelihood ratio.

On the chi-square test, it was found that only daily study hours (during normal days), residence (day scholars), library study, morning study among study timings, active recall and MCQS solving among study strategies, and watching online videos among study sources made a significant association with academic performance i.e., P < 0.05

Pearson’s correlation showed that age, daily study hours (during the exam), and study location were negatively correlated with academic performance, this correlation was statistically non-significant (p > 0.05). Only daily study hours (during normal days) and study strategies made a statistically significant positive correlation (p < 0.05) with academic performance, (Table-III).

**Table-III:** Correlation analysis of academic performance with its associated factors.

Correlations									

		PLFE	Age	DSH(NR)	DSH (E)	SL	ST	SST	SSO
PLFE	Pearson Correlation		-.156	.501^**^	-.126	-.091	.135	.188^*^	.099
Age	Pearson Correlation			.052	.321^**^	-.018	-.008	.111	-.067
DSH(NR)	Pearson Correlation				-.064	-.029	-.055	.093	-.038
DSH (E)	Pearson Correlation					.097	-.035	.000	-.015
SL	Pearson Correlation						.039	.064	-.006
ST	Pearson Correlation							.273^**^	.152
SST	Pearson Correlation								.406^**^
SSO	Pearson Correlation								

** . Correlation is significant at the 0.01 level (2-tailed).,

* . Correlation is significant at the 0.05 level (2-tailed)., DSH(NR) =Daily study hours (normal routine), DSH(E)=Daily study hours (exam days), SL=Study location ST=Study timings, SST=Study strategies, SSO=Study sources, PLFE=Percentage in last professional exam.

The multiple regression model showed that only age and daily study hours (during normal days) among the independent variables had a significant predictability for the dependent variable of high academic performance, F (6,131) =9.596, (p < 0.05). Moreover, the adjusted R square =0.300 depicts that the model explains 30.5% of the variance in academic performance, (Table-IV).

**Table-IV:** Multiple regression analysis of academic performance with associated factors.

Associated variables	Unstandardized Coefficients		Standardized Coefficients	t	P-value

	B	SE	Beta		
(Constant)	4.021	0.742		5.422	0.000
Age	-0.079	0.033	-0.186	-2.423	0.017
DSH(NR)^a^	0.484	0.07	0.504	6.944	0.000
DSH(E)^b^	-0.018	0.067	-0.02	-0.264	0.792
Study Location	-0.132	0.106	-0.09	-1.247	0.215
Study Timings	0.118	0.07	0.127	1.693	0.093
Study Strategies	0.07	0.049	0.117	1.422	0.157
Study Sources	0.03	0.01	0.039	0.487	0.627

Dependent Variable: Percentage in last professional exam, R2 = 0.335, F (6, 131) = 9.596.

## DISCUSSION

Our study revealed that daily study hours during normal routine, residence, studying at the library, studying in the morning, using active recall, MCQs-based study strategies, and watching online videos had a significant association with academic performance. In our study majority of students (53.6%), had average scores of 70-80%. A study conducted by Saber et al. also showed that the majority of students (51%) had average academic performance.^7^ This showed that the majority of the students of an institute have average grades.

In a previous study conducted at Ayub Medical College Abbottabad, only 9.1% of medical students were active learners with no significant correlation with academic scores^8^, compared to our study where we found a significant relationship between active recall and academic performance. Studies show that individuals who study more hours per day score better;^9^ However, there is a stage beyond which more study hours no longer help.^10^ Poor time management is the main reason for poor performance,^11^ with preclinical students and day scholars having better time management skills compared to clinical students and hostilities respectively.^12^

The results of Ahmed et al. are consistent with our study results in the association between gender and study habits.^13^ The presence of an equal educational environment for both genders are a possible reason. We believe that every student, regardless of gender, should have knowledge of study skills and apply them. Yet another study conducted on university students of KP, Pakistan shows that the female gender was associated with better academic achievements.^14^

MCQS solving/problem solving based approach also leads to better academic performance, a similar study was conducted among second-year medical students using study aids and exam performance compared to those who don’t use study aids which yielded results similar to our study.^15^ Another study in the US indicated that the number of practice test items correlated to higher scores in the national licensure exams.^16^

Our study shows students who used the library were more likely to succeed academically, as reported by Sami A that factor such as the library environment, availability of resources, and responsive staff greatly influence students’ study habits and academic outcomes.^17^ Jan SU et al. state that individuals with high emotional quotient visit the library more often and have high grades.^18^

Our study also pursued the idea that where people lived had a significant impact on the academic performance of the student, with day scholars achieving better grades on average compared to hostellers. According to a study conducted in the Northwest School of Medicine, the most common motivation amongst the average and good-scoring students was the family pressure^19^, showing that the closer you are to your family the more motivated and concerned you will be about your studies.

According to various studies, students who study in the morning tend to achieve better academic results. A study at Middle Tennessee State University found that male students who took afternoon classes earned lower grades than those who took morning classes.^20^ Contradictory to these certain studies reported that early wake-up in the morning and morning classes are associated with low grades, one of the reasons being sleep deprivation.^21^

Online videos are also a good source for learning and those who use them have better academic performance, one of the studies conducted on the students of Karachi University shows that students prefer to use video sources specifically YouTube for learning topics through animation and simulation^22^, indicating the emergence of online video sources as an important tool for academic achievers. So, students need to consider proper study habits, timings, strategies, and sources to achieve better academic outcomes. This study adds significant contributions to the medical literature in terms of providing research-based strategies and methods to help medical students improve their academic outcomes.

### Limitations:

It was a cross-sectional study, so we cannot establish a causal relationship between various factors and academic performance. Only MBBS students of Peshawar, excluding other programs, were included in this study which may limit the generalizability of the results. Our study did not consider some of the important factors like socioeconomic status and mental health. Longitudinal studies, considering all factors will be needed to establish the causal relationship.

## CONCLUSION

This study aimed to determine the association between study habits, study timings, study strategies, and study sources with academic performance in medical colleges in Peshawar**,** Pakistan**.** The study found that daily study hours during a normal routine, day scholars (residence), studying at the library, morning time study, using active recall, and MCQs-based study strategies, and watching online videos had a significant association with academic performance.

### Author`s Contribution:

**MO:** Conceived, designed, analyzed, and drafted the article, revised, final approval.

**AS**: Designed, collected, revised, final approval.

**RUH:** Designed, analyzed, and collected the data, revised, final approval.

**MAB**: Designed, collected the data, analyzed, revised, and drafted the article, final approval.

All authors have read approved the final version and are responsible for all aspects of the work.
